# Factors Affecting Remote Workers’ Job Satisfaction in Utah: An Exploratory Study

**DOI:** 10.3390/ijerph20095736

**Published:** 2023-05-06

**Authors:** Amanda D. Ali, Lendel K. Narine, Paul A. Hill, Dominic C. Bria

**Affiliations:** 1 Home and Community Department, College of Agriculture and Applied Sciences, Utah State University, Logan, UT 84322, USA; paul.hill@usu.edu (P.A.H.); dominic.bria@usu.edu (D.C.B.); 2 Youth Programs Department, College of Agriculture and Applied Sciences, Utah State University, Logan, UT 84322, USA; lendel.narine@usu.edu

**Keywords:** AMO framework, employee wellbeing, human resources bundles, job performance, job satisfaction, organizational culture, remote worker, Utah

## Abstract

With structural changes in work arrangements, employee retention becomes more important for organizational success. Guided by the Ability, Motivation, Opportunity (AMO) framework, this study investigated the factors affecting remote workers’ job satisfaction and personal wellbeing in Utah. From a sample of *n* = 143 remote workers, the study used a correlational design to identify the significant predictors of job satisfaction and personal wellbeing. It mapped the relationships between significant predictors of job satisfaction and personal wellbeing and explored the role of human resources (HR) policies and organizational culture in a remote work environment. Results showed intrinsic motivation, affective commitment, opportunity, and amotivation affected employee job satisfaction, while self-efficacy, amotivation, and job satisfaction affected personal wellbeing. A structural equation model (SEM) showed that remote workers with higher levels of self-efficacy, lower amotivation, and higher job satisfaction were likely to have greater personal wellbeing compared to others. When exploring the role of HR, findings showed that HR bundles and organizational culture indirectly affected job satisfaction but had a direct effect on the most important predictors of job satisfaction and personal wellbeing. Overall, results demonstrated the interconnectivity of HR practices, AMO factors, job satisfaction, and personal wellbeing.

## 1. Introduction, Purpose, and Objectives

Job satisfaction is often linked to elements of job performance and productivity. According to [1], job satisfaction is “the pleasurable emotional state resulting from the appraisal of one’s job as achieving or facilitating the achievement of one’s job values” (p. 316). It is further described as the value responses (or fulfillment) an individual experiences when engaging in a task compared to their perceived standard of measure [1,2]. In human resources management (HRM), job satisfaction is a key component of one’s motivation to perform [3]. Performance is defined as “the total expected value to the organization of the discrete behavioral episodes that an individual carries out over a standard period of time” [4]. It pertains to results that influence one’s behavior over time that either contribute to or deter from expected organizational goals. When employees experience a sense of achievement related to work tasks (i.e., job satisfaction), their organizational behaviors (or performance) tend to be positive [5].

With structural changes in work arrangements, employee retention becomes more important for organizational success. In March 2020, the World Health Organization declared COVID-19 a pandemic [6]. The United States (U.S.), along with many countries, ordered schools and businesses to close and declared worldwide travel restrictions. These policies changed the way people interacted with one another; social distancing and isolation meant few people were gathering in public places. Such actions were necessary to slow the spread of COVID-19. In the U.S., many states required only essential services and businesses (e.g., healthcare, pharmacies, etc.) to remain open. For businesses considered non-essential, their options were to adapt to a changing work environment or permanently close. About 41% of businesses reported a temporarily closed status due to the pandemic, with permanent closures of around 2% [7,8]. Associated with these closures was an increase in employee attrition, with a 39% total reduction in part-time and full-time employees [7]. 

As the nature of work operations changed [9], the nature of tasks also changed. For many businesses, compatible work tasks were completed primarily online, as employees worked outside the typical office environment, with most working from home. Given this change in the work environment, professional and personal duties tended to overlap. One study found that nonwork (personal) responsibilities predicted various types of nonwork-related interruptions in the workday [9]. Furthermore, nonwork distractions, breaks, and intrusions had the strongest negative relationship with performance, while distractions and intrusions strongly predicted emotional exhaustion. Nonwork intrusions were defined as a “reluctance to switch attention and the preference for staying focused on the initial/interrupted task” [9]. Interestingly, frequent breaks also contributed to fragmented schedules, as they potentially disrupted performance due to family interference, causing delays in work tasks and increased levels of stress. A meta-analysis review of the Jobs-Demands Resources (JD-R) Theory also identified that job engagement was highly correlated with job satisfaction and commitment [10]. During the pandemic, work and family conflict negatively affected workplace wellbeing for those working from home [11]. That is, as family interference with work increased, there was a decrease in workplace wellbeing. Work and family conflict referred to incompatible work (professional) and family (personal) duties, while workplace wellbeing was defined as employee job satisfaction.

Other studies focusing on employee wellbeing found work–life balance, isolation, loneliness, lack of boundaries between work and family, home-office constraints, uncertainty in work tasks, inadequate tools, procrastination, and effective communication were major challenges facing remote employees [12]). These findings are also consistent with the JD-R meta-analysis review which identifies both work demands and personal responsibilities as influencing employee wellbeing [10]. Specifically, the review identified that work demands (e.g., unclear roles, job task conflicts, and workload) caused exhaustion and burnout among some employees [10]. To help ease some of these challenges, different approaches to work were proposed in the literature to increase job satisfaction and employee wellbeing. For example, managers can redesign work tasks to suit a remote environment or consider the level of autonomy required for tasks and the degree of interruption that may occur when working remotely [11]. Remote employees were enthusiastic about their work tasks, valuing the autonomy and flexibility that working remotely offered as it related to positive wellbeing [12]. A recent study found that a hybrid work arrangement fostered increased self-assessed productivity and work satisfaction [13]. In Bloom et al.’s study, specific tasks were designed for in-office (e.g., team meetings, training) and at-home (e.g., individual tasks, reading) environments [13].

Managing these challenges is important for the future of remote work success. According to [14], a Gartner survey reported 82% of business leaders planned to continue offering remote work arrangements, either fully remote or hybrid, post-pandemic. Another study found that remote work arrangements will continue post-pandemic, with a projected 20% of fully remote employees in the workforce [15]. In this study, many workers were also willing to continue hybrid remote positions at the expense of a reduced salary. In a study of Polish employees and employers’ remote work preferences, employees would, on average, sacrifice 5.1% of their income to have a hybrid work-from-home option instead of a fully remote option [16]. Another study investigated the increase in working from home across 27 countries [17]. They found that workers wanted an average of 1.7 work-from-home days per week and valued a hybrid option for 2–3 days per week at 5% less than their current pay. These recent examples demonstrate a shift in the mindset and patterns of employees and organizations with respect to remote work. As such, this study sought to explore the organizational and personal factors related to the job satisfaction and wellbeing of remote workers in Utah.

Given the importance of employee job satisfaction and wellbeing as it relates to organizational performance in a remote working environment [18,19], this study adapted the Ability, Motivation, Opportunity framework to explore factors impacting job satisfaction and personal wellbeing. Specifically, this research sought to explore individual and organizational factors affecting the job satisfaction and personal wellbeing of remote employees. Objectives were to Identify the significant predictors of job satisfaction; Identify the significant predictors of personal wellbeing; Map the relationships between significant predictors of job satisfaction and personal wellbeing; Explore the role of human resource (HR) policies and organizational culture in a remote work environment.

## 2. Literature Review

### 2.1. Work Environments

Despite the work environment (i.e., onsite, remote, or hybrid), employees are at the center of all workday operations. With rapid technological changes and dynamic shifts in work cultures and environments, employee retention is imperative to organizational success [20]. Scholars found that employee turnover in steel mini mills was lower in commitment-type HR systems as opposed to control-type HR systems [21]. Both these systems were differentiated by various organizational conditions that influenced employee behavior [21]. Control HR systems are characterized by a reward structure, where employee rewards are measured against an output criterion. This structure aims to reduce labor costs and improve efficiency via employee compliance [22,23]. Characteristics under this system include incentives with limited benefits; narrow job descriptions; little communication, socialization, and employee involvement in managerial decisions; no grievance procedures; low skill requirements and wages; intense supervision; and limited training [22].

Commitment HR systems focus on establishing “psychological links between organizational and employee goals” to develop positive attitudes and behaviors [21]. The aim is to create employee commitment, trusting that they will complete work tasks in accordance with organizational goals [21]. Specific characteristics under this system include salaries with extensive benefits, broad job descriptions, high employee involvement, dispute resolution procedures, shared organizational information with employees, high skill requirements, self-directed teams, and skills training [22]. Commitment HR systems are present in certain employee–organization approaches. Employee–organization relationships are strategies that focus on the employer’s perception of the working relationship with employees (i.e., employer expectations and employee incentives that achieve desired organizational outcomes) [24]. One review also found that altering work environments or providing employees the autonomy to influence their work environments could help foster positive engagement in work tasks [10].

When analyzed against other types of employee–organization relationships, employees’ attitudes and performance were more favorable toward a mutual or balanced type of relationship [25]. As such, managerial involvement appears to play a vital role in employee satisfaction, motivation, and performance under certain circumstances. Employees’ motivation likely increased with motivation- and opportunity-enhancing HR practices [26]. They defined employee motivation as effort, “manifested by positive work attitudes (e.g., collective job satisfaction, commitment, perceived organizational support), and work behaviors e.g., organizational citizenship behavior” [26]. Based on their findings, employees are likely to perform better when they are highly knowledgeable about their work tasks and are given the opportunity to contribute to organizational goals (as supported by those HR practices). They further found an association between investments in skills/ability, motivation, and employee involvement in organizational HR practices and financial goals. That is, it is beneficial for organizations to retain skilled employees and meet organizational goals by investing in HR practices that support employee motivation and development.

Employee performance tends to be more positive when their organization is willing to commit to them on a long-term basis [25]. Part of this commitment stems from the social exchange between employer and employee. Social exchange is defined as “the exchange of activity, tangible or intangible, and more or less rewarding or costly, between at least two persons” [27]. For organizations, this relationship exists between employer and employee and extends outside short-term monetary rewards to employees [25]. It exists as the interaction between both parties, where the employer considers an employee’s wellbeing and career, while the employee reciprocates with obligations and contributions to the organization (e.g., exceeding expectations and accepting tasks outside regular agreements). As such, employee behaviors or attitudes (performance) are more favorable toward achieving organizational goals.

### 2.2. Work Redesign

Work environments can be considered dynamic; they are ever-changing and adapt to technological trends, organizational goals, and/or global events. For example, there was a major upset to work environments globally during the coronavirus pandemic in 2020. Since isolation and distancing oneself from others were key behaviors in reducing the spread of the virus, working from home became a solution for business continuity and job retention. While remote work is not a new concept, many organizations transitioned into this type of work arrangement for the first time to slow the spread of the pandemic [28,29]. While the practice has been around since the early 1960s, its popularity grew in the early 1970s. The term telecommuting referred to “computational and telecommunications components which enable employees of large organizations to work in offices close to (not generally *in*) their homes, rather than commute long distances to a central office” [30]. This definition relates to an early form of what is now considered virtual work. 

At the core of a remote work system are employees and their ability to perform work tasks in a changing environment. Since organizations are dependent on productive workdays to meet daily and organizational goals, it is essential to ensure employees are well-equipped to undertake their work tasks in alternative work arrangements. In some cases, this includes redesigning work tasks based on *how* they work, but it also extends to rewards or organizational benefits. The term ‘work redesign’, are “activities that involve the alteration of specific jobs (or systems of jobs) with the intent of improving both productivity and the quality of employee work experiences” [31]. Usually, tasks can be redesigned within specific criteria to allow for productivity and efficiency. For instance, they can be redesigned to include motivating and satisfying work for independent employees, or autonomy within teams designated for large segments of work [10,31]. 

While there is no consensus on all the attributes of a properly redesigned job, common traits include self-directedness opportunities, added responsibilities, and decision-making regarding workflow [31]. However, when work tasks are enriched (or perceived as meaningful by employees) and effectively redesigned to improve autonomy, skill diversity, task identity and significance, and job feedback [32], it is likely that employee satisfaction, performance, and motivation will increase [31]. Specifically, opportunities for personal growth and development, as well as internal motivation, were found to be strongly correlated with employee satisfaction, more so than organizational characteristics [31]. As purported, human resource practices, motivation, opportunities for growth and development, and training to enhance work skills collectively play a vital role in overall job satisfaction and performance. However, as the nature of work continues to change (*how* and *where*), employee satisfaction is pushed to the forefront as a key component of business continuity and organizational success.

While there is increased literature on remote employees’ job satisfaction [33,34,35], several studies have also investigated the interaction between remote work and personal wellbeing. One study found remote workers experienced increased social isolation and stress [36], while another discussed the negative effects of remote work on work–life balance [37]. Nonwork interruptions, an unbalanced work and family dynamic, isolation, and loneliness are all concerns that may persist in the future of remote work arrangements, potentially leading to burnout, stress, and employee turnover [38]. A study by Qualtrics indicated HR managers should be aware of employee wellbeing; results indicated 61% of remote employees will not take a sick day because of a heavy workload [39]. Therefore, providing remote employees with a supportive work environment could help increase their job satisfaction and personal wellbeing, which are critical factors for employee performance and ultimately organizational success.

### 2.3. Ability, Motivation, Opportunity Framework

The Ability, Motivation, Opportunity (AMO) framework guided this study. It was first credited to [40], and further developed by [41]. The AMO framework articulates a relationship between employee performance and HRM, considering the role of three variables (i.e., ability, motivation, opportunity) on employee performance [42]. While various industrial psychology scholars have contributed to the literature on employee performance, early compositions focused on personal characteristics. Other works have focused on motivation, skills, and training as predictors of performance. However, the “emphasis on predicting job performance, and particularly productivity rests upon the acceptance of certain values” [43]. In other words, productivity can be directly related to outputs that have economic value to the organization. Other antecedents of performance include personality, leadership, and organizational design [44]. As such, many studies have sought to investigate factors affecting productivity and/or job performance. 

According to some psychologists, selection, placement, and training determine performance, while others support motivational characteristics as an antecedent to job performance [44]. However, scholarly works supported past studies that posited an interactive relationship between ability and motivation [45,46]. Later works on employee–employer relationships reported that an external component was necessary for explaining employee behaviors: the operational work environment. In study the authors maintained that participation in the workplace was an important but missing factor in performance. They asserted *opportunity* to participate accounted for the external environment and it accounted for variables outside the control of employees that could affect performance. As such, “even though an individual may be willing, and have the capacity to engage in a given behavioral act, whether or not this act can be consummated depends on the presence and arrangement of facts in the person’s objective environment” [44]. Opportunity, along with ability and motivation, interact to explain job performance [44].

Ability, or capacity to perform, represents “the physiological and cognitive capabilities that enable an individual to perform a task effectively” [44]. Facets of capacity to perform are “ability, age, knowledge, skills, education, intelligence, endurance, stamina, energy level, and motor skills” [44]. Motivation, or willingness to perform, is defined as the “psychological and emotional characteristics that influence the degree to which an individual is inclined to perform a task” [44]. Factors under this dimension include “motivation, job satisfaction, job status, anxiety, legitimacy of participation, attitude, perceived task characteristics, job involvement, ego involvement, self-image, personality, norms, values, perceived role expectations, and feelings of equity” [44]. Opportunity “consists of the particular configuration of the field of forces surrounding a person and his or her task that enables or constrains that person’s task performance and that are beyond the person’s direct control” [44]. This empowers employee performance to meet organizational goals. Factors influencing opportunity to perform include “tools, equipment, materials and supplies, working conditions, actions of co-workers, leader behavior, mentorism, organizational policies, rules and procedures, information, time, and pay” [44]. 

After several iterations, the AMO framework considered a high-performance work system (HPWS) and suggested when employees are capable, motivated, and can participate in their work environment, they will perform better because the work environment facilitates their job [47,48]. An effective HPWS ensures “employees have the opportunity to contribute discretionary effort” [49], they have the skills and incentives to perform, and they benefit from enhancing practices that support these dimensions of performance [49,50]. HRM is defined as “involving management decisions related to policies and practices which together shape the employment relationship and aim at achieving individual, organizational, and societal goals” [51]. One of HRM’s responsibilities is creating a HPWS that lends itself toward organizational goal accomplishment and sustained competitiveness, in which human resource (HR) practices foster the three (AMO) dimensions of performance [47,50,51]. 

Practices that enhance AMO dimensions are called high-performance work practices (HPWPs). In an integrated literature review [48], practices under each component of performance were summarized. They found a consensus among many studies that ability-enhancing practices constituted training and career, as well as recruitment and selection. While performance evaluation practices were referenced, they were not as common. Motivation-enhancing practices were divided into extrinsic and intrinsic motivation. Practices relating to extrinsic motivation were referenced in most studies of the integrated literature review [48]. The top practices under extrinsic motivation were performance appraisal and extrinsic incentives. Less common practices included individual pay for performance, group-level pay for performance, recognition, job security, internal promotion, social activities, and work–life balance opportunities. The least common practices under intrinsic motivation were motivation to learn, personal or team satisfaction, willingness to perform, corporate sense, and collaborative climate.

Finally, opportunity-enhancing practices were classed into four groups or bundles [48]. These were employee involvement, knowledge-sharing, job design, and decision making. The most mentioned practices under employee involvement included teamwork, problem-solving teams, and self-directed teams. Quality circles were the least mentioned in research articles. The most common knowledge-sharing practice was information sharing and communication, and less common was suggestion systems, complaint systems, or surveys. Job design practices included job description; less common were support from HR, job rotation, level of internationalization, and favorable work conditions. Decision-making practices included autonomy and, to a lesser extent, irregular and regular flexibility [48]. 

As synergy is at the core of HRM systems, bundles of practices (i.e., HPWP) within a HPWS are thought to produce the greatest effect on performance [52,53]. Synergy can be defined as “positive outcomes deriving from the interrelations among a system’s components” [52]. It is assumed that synergies could exist between organizational work and high-performance work practices (HPWPs) if applied collectively within a system, not separately [52,54]. In their study [53], HPWPs enhanced performance, accounting for 20% of the variation in organizational performance. This relationship was stronger within HPWS. As HPWPs increase employees’ knowledge, skills, and abilities, their motivation to perform also increases [55]. Collectively, these activities result in higher job satisfaction, productivity, and performance, as employees make better decisions [53]. Although many studies support synergistic effects, there is no consensus on the HPWPs that should be included in a HPWS [48].

## 3. Materials and Methods

### 3.1. Population and Sample

This study used a correlational design as discussed by [56], and sample data was gathered from a population of remote workers in Utah. Study participants met two parameters for inclusion in the study: (a) they were fully remote employees or had a hybrid work arrangement, and (b) they were employed full-time. A convenience (non-probability) sampling approach was used to gather data from the population, and the final sample size consisted of 143 respondents (*n* = 143). After exemption from Utah State University Institutional Review Board, Centiment^®^ was contracted to gather data from the population using a researcher-developed, closed-ended, online questionnaire. Centiment^®^ surveyed individuals from their existing opt-in panels that met the inclusion parameters, and data collection was completed in May 2022. 

With respect to sample characteristics, 12% of respondents were between 18 and 24 years old (*n* = 17), 34% were 25–34 years old (*n* = 49), 27% were 35–44 years old (*n* = 38), 12% were 45–54 years old (*n* = 18), 12% were 55–64 years old (*n* = 17), and 3% were 65 years or older (*n* = 4). Most respondents worked in the private sector (67%, *n* = 96), while 20% worked in the public sector (*n* = 28) and 13% worked in not-for-profit organizations (*n* = 19). The top three industries represented by respondents in the sample were healthcare (17%, *n* = 24), professional, scientific, or technical services (16%, *n* = 23), and finance or insurance (13%, *n* = 19). Most remote employees in the sample worked for large organizations with 1000 or more employees (57%, *n* = 81). Lastly, respondents had varying years of experience, but most had less than 4 years of experience at their organization (58%, *n* = 83). While our target population included both hybrid and remote workers, most respondents were fully remote employees (63%, *n* = 90). A series of Chi-square analyses was conducted to examine any differences in respondents’ characteristics by their working arrangement (i.e., remote vs. hybrid); there were no statistically significant differences in age, organizational sector, industry type, organization size, or experience based on working arrangement. 

There are limitations to the study design, including (a) generalizability of results due to the sampling approach and sample size and (b) conclusions on causation. First, the use of a non-probability sampling approach limits the external validity of the results. The minimum sample size was estimated in G*Power. Using a standard significance of α = 0.05 and power = 0.80 (80%), the minimum sample size needed for a medium (or stable) effect size was *n* = 160 in a multiple linear regression with 21 predictors. With a sample of 143, the power was 75%. While 80% power is considered ideal, a lower power does not disqualify the results, as researchers should factor the cost limitations of attaining a larger sample [57]. As a result, we applied several measures to improve the accuracy of estimates from the multivariate techniques as discussed in later sections. Regardless, caution is recommended when generalizing the findings beyond the sample of remote workers. With respect to causation, the correlational (non-experimental) design of this study prohibits the authors from concluding any cause-and-effect relationships. The authors recommend readers explore the results and determine their appropriateness to the context.

### 3.2. Instrument Design

The Tailored Design Method was used to develop the study’s questionnaire [58]. We adopted best practices for crafting closed-ended questions (e.g., grouping of items within constructs, no double-barreled items, exhaustive response options, and clearly labeled anchors). All items in the questionnaire were adapted from the HRM and AMO literature and aligned with our theoretical framework. Upon creating a draft questionnaire, we sought feedback from an expert panel for face validity. The expert panel consisted of individuals with expertise in evaluation, survey development, social science research methods, remote work education, and human resources. We conducted a pilot test with 20 individuals from the target population. No changes were made to the questionnaire after the pilot test. The final survey consisted of 141 items, and respondents took approximately 20 minutes to complete the survey. Since we used a panel provider to gather data, there were no missing responses in the dataset.

### 3.3. Description of Study Constructs

Table A1 provides a summary of all constructs and matching domains examined in this study (see Appendix A). Constructs were measured on Likert-type scales and semantic differential scales. Construct scores were created from individuals’ responses to corresponding items.

### 3.4. Summary Statistics of Constructs

Table 1 provides a descriptive summary of all domains and corresponding constructs assessed in this study. While all constructs were adapted from existing measures as outlined in the methods, we performed an exploratory factor analysis (EFA) followed by a confirmatory factor analysis (CFA) to examine the communalities among items in each construct. This process was effective for (a) identifying items with low extraction commonalities and factor loadings (of <0.49) and (b) ensuring each construct represented a unique solution based on eigenvalues (of >1). The CFA process led to the omission of one item in “Opportunity” and one item from “Internal Mobility.” The process led to the restructuring of “Internal Reward” to create a new construct for “Motivation-enhancing” in HR-Bundles, and the merging of “Training” and “Ability-Enhancing.” The final constructs shown in Table 1 were unique solutions from the CFA, and there were no latent variables (sub-constructs) within each construct. After the CFA, we examined each construct for reliability and any major violations of normality.

Cronbach’s alpha measure of internal consistency is widely used as a proxy to assess construct reliability [59,60]. While there are many “acceptable” ranges, a generally safe assumption is a Cronbach’s alpha greater than 0.70 indicates acceptable internal consistency. An acceptable Cronbach’s alpha suggests the operational construct provides a reliable index of the theoretical construct [61,62]. For normality, the literature provides many cutoff values for skewness and kurtosis when assessing normality or the potentially harmful effect of non-normally distributed data in multiple regression and Structural Equation Models (SEMs). However, a recurring cutoff is an absolute value of two (|2|) for skewness, and three (|3|) for excess kurtosis to assume an asymptotically normal distribution, or more importantly, a lowered effect of Type 1 error due to non-normality [63,64,65,66,67]. From Table 1, Cronbach’s alpha indicates all constructs had an acceptable level of internal consistency. 

Both skewness and excess kurtosis estimates for all constructs were below the cutoffs of |2| and |3| respectively. Therefore, we assumed the constructs were reliable and did not violate the assumptions of normality to the extent of causing biased estimates in the Ordinary Least Squares (OLS) or SEM. As discussed below, we took additional steps to prevent biased estimates in the multivariate models. Table 1 also shows relatively moderate to high mean scores across most constructs based on the minimum and maximum ranges. For example, remote workers, on average, rated their ability (i.e., self-efficacy) as high (*M* = 82.64; *SD* = 15.77) on a scale ranging from 1 to 100. Meanwhile, the mean score for amotivation was low (*M* = 2.07; *SD* = 1.02) on a 5-point scale, which agrees with the moderate to high mean scores for intrinsic motivation and identified regulation. 

However, external regulation, which refers to external motivation to avoid undesirable outcomes, was also moderate to high on the 5-point scale (*M* = 3.76; *SD* = 0.94). Together, mean scores for the Motivation domain indicated remote workers had generally moderate to high levels of motivation in their jobs, but the source of their motivation varied between internal and external factors. Another noteworthy finding from the descriptive results was related to organizational commitment. The sample had moderate levels of affective (*M* = 4.79, *SD* = 1.63) and normative commitment (*M* = 4.07, *SD* = 1.44) on the 7-point scale, while having higher levels of continuance commitment (*M* = 5.52, *SD* = 1.08). “Employees with strong affective commitment remain because *they want to*, those with strong continuance commitment because *they need to*, and those with strong normative commitment because they feel *they ought to do so*” [68]. Remote workers in our sample felt a greater level of continuance commitment compared to affective and normative commitment, suggesting many stayed in their jobs because they needed to. With respect to the two outcome (or dependent) variables of importance in this study, on average, there was a moderate level of job satisfaction and personal wellbeing among respondents. Refer to Table A1 for a description of each construct to assist with the interpretation of mean scores shown in Table 1.

### 3.5. Data Analysis

This study explored the individual and organizational factors affecting job satisfaction (Outcome 1) and personal wellbeing (Outcome 2). As shown in Table A1 and Table 1, a review of the literature coupled with the AMO framework provided 19 independent factors. We used an OLS regression to address objectives (a) and (b), an SEM to address objective (c), and zero-order Pearson’s correlations to address objective (d). For the OLS regression models, we estimated bootstrap confidence intervals (BC) to assess the precision of the regression estimates [69]. As a resampling procedure, bootstrapping is effective when using imperfect datasets, as it improves the accuracy of the predictors [70]. However, our ability to generalize the results to a population of remote workers in Utah remains limited. Compared to standard OLS, the bootstrapping procedure is more robust to non-normality and heteroscedasticity [71]. Therefore, we opted for bootstrapping as a conservative approach to avoid biased predictors [72,73].

Guided by the AMO framework, the SEM was primarily used as an exploratory approach to address objective (c); it allowed us to map the relationship between statistically significant explanatory constructs from the OLS models and the two outcome variables of focus in this study. The SEM is commonly used to investigate complex relationships between constructs [66]; it was appropriate for understanding the covariance between constructs and their effect on job satisfaction and personal wellbeing. Lastly, we utilized Pearson’s correlations to explore the role of HR bundles and organizational culture in a remote work environment for objective (d). All analyses were conducted on verified unidimensional constructs from the initial CFA, and respondents’ income level was statistically held constant in the OLS models. Data were analyzed using SPSS^®^ (28, IBM, New York, NY, USA) with AMOS^®^ (26, IBM, New York). 

## 4. Results

### 4.1. Objective (a): Predictors of Job Satisfaction

Table 2 shows the first OLS regression model for job satisfaction (Outcome 1). The bootstrapped regression model in Table 2 was statistically significant (*F* = 20.88_20, 122_; *p* < 0.001), suggesting the independent variables (taken together) had a reliable level of explanatory power on job satisfaction. The adjusted R-squared was 0.74. This indicated the independent variables explained 74% of the variance in job satisfaction. For the independent coefficients (β) shown in Table 2, results indicated intrinsic motivation, amotivation, opportunity, and affective commitment had a statistically significant effect on job satisfaction. Intrinsic motivation had a statistically significant and positive effect on job satisfaction (*p* < 0.001). When remote employees experienced pleasure in their tasks and found them to be exciting or interesting, they experienced a greater level of overall satisfaction in their jobs compared to others who did not. Intrinsically motivated employees performed their tasks because they found them interesting, not because they sought external rewards.

In contrast, results showed a statistically significant and negative relationship between amotivation and job satisfaction (*p* < 0.001). Remote workers who do not see purpose or value in their roles experienced a lower level of overall job satisfaction compared to others who did not. A motivated employees may feel helpless and are mainly performing their tasks for external rewards (i.e., external regulation). Opportunity had a statistically significant and positive effect on job satisfaction (*p* < 0.05). When employees believed they were involved in the organizational decision-making process, they were more satisfied with their jobs compared to others who were not. Opportunity is a major component of the AMO model; a higher degree of opportunity is reflected by a decrease in the “distance” between management and employees. Remote employees with the opportunity to participate will feel like they have a voice in the organization. 

Lastly, affective commitment had a statistically significant and positive effect on job satisfaction (*p* < 0.001). When remote workers felt a greater emotional attachment and personal connection to the goals and values of the organization, they were more likely to experience a greater level of overall satisfaction with their jobs compared to others who did not. Employees with strong affective commitment to an organization are likely to remain in the job because *they want to*, not because *they must* (i.e., affective vs. continuance commitment).

### 4.2. Objective (b): Predictors of Personal Wellbeing

Table 3 shows the results of a bootstrapped OLS regression to explain the factors affecting personal wellbeing (i.e., Outcome 2). This model included job satisfaction as an explanatory variable together with the other factors in Table 2. As previously discussed, bootstrapping was used as a conservative approach to avoid biased estimates and improve model accuracy. The bootstrapped regression model in Table 3 was statistically significant (*F* = 5.61_21, 121_; *p* < 0.001), suggesting the (20) independent variables had a reliable level of explanatory power for personal wellbeing. In addition, the adjusted R-squared was 0.41, indicating the explanatory variables explained 41% of the variance in personal wellbeing. As expected, the regression for job satisfaction in Table 2 had more explanatory power compared to the regression for personal wellbeing. Personal wellbeing is a multifaceted construct and is likely to be dependent on factors external to one’s formal employment [74].

Results indicated self-efficacy, amotivation, and job satisfaction had a statistically significant effect on personal wellbeing. While self-efficacy was not influential on job satisfaction (as shown in Table 2), it had a statistically significant and positive effect on personal wellbeing (*p* < 0.001). This indicates that when remote employees were confident in their ability or had higher levels of self-esteem, they experienced higher levels of personal wellbeing compared to others who did not. Amotivation had a statistically significant and negative effect on personal wellbeing (*p* < 0.05). Amotivation extends to employees’ value or purpose in their job. It appeared when remote employees had a lowered sense of purpose in their job; it had an adverse effect on personal wellbeing. Lastly, job satisfaction had a statistically significant and positive effect on personal wellbeing (*p* < 0.001). As remote employees experienced greater job satisfaction, they were likely to have higher levels of personal wellbeing compared to others.

### 4.3. Objective (c): Mapping Predictors of Job Satisfaction and Personal Wellbeing

**Figure 1:**
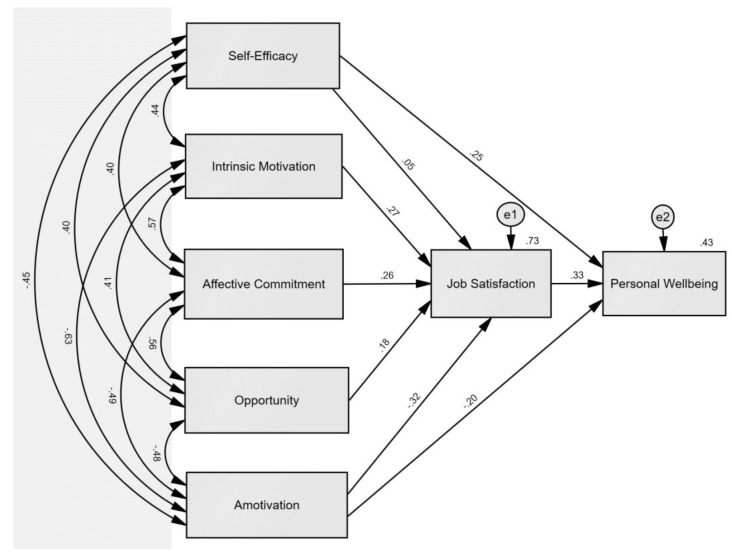
SEM illustrating the Standardized Coefficients between Significant Constructs.

Figure 1 provides a summary of the results from the SEM. With evidence from Table 2, we identified the significant role of intrinsic motivation, amotivation, opportunity, and affective commitment on job satisfaction. In Table 3, we established the effect of self-efficacy, amotivation, and job satisfaction on personal wellbeing. Taken together, findings from the two bootstrapped OLS models informed the SEM. The literature indicates a good model fit based on data can be assumed with (a) a non-significant Chi-square test, (b) a Comparative Fit Index (CFI) and Tucker Lewis Index (TLI) larger than 0.95, and (c) a Root Mean Square Error of Approximation (RMSEA) of less than 0.08 [64,75,76,77]. For the SEM illustrated in Figure 1, the model proved to be a good fit: (a) the Chi-square test statistic was not significant (*X*^2^ = 5.28_3_, *p* = 0.12), (b) CFI = 0.99; TLI = 0.96, and (c) RMSEA = 0.08. While the RMSEA was borderline, the SEM met the indices of good fit as suggested in the literature. The five explanatory constructs on the left side of Figure 1 (i.e., self-efficacy, intrinsic motivation, affective commitment, opportunity, and amotivation) accounted for 73% of the variance in job satisfaction. Then, the explanatory variables including job satisfaction explained 43% of the variance in personal wellbeing. The SEM shows the direct effects of each construct on job satisfaction and personal wellbeing and the covariances between explanatory constructs.

Figure 1 shows the standardized coefficients between constructs in the SEM. All direct relationships were statistically significant, except for self-efficacy on job satisfaction (*β* = 0.05, *p* = 0.38). Based on the standardized coefficients, amotivation had the greatest effect on job satisfaction (*β* = −0.32, *p* < 0.001), followed by intrinsic motivation (*β* = 0.27, *p* < 0.001), affective commitment (*β* = −0.32, *p* < 0.001), and opportunity (*β* = −0.32, *p* < 0.01). These results suggest remote employees who saw purpose or value in their work tasks (low amotivation), experienced pleasure or excitement in their roles (high intrinsic motivation), felt like they had an emotional attachment and personal connection to the organization (high affective commitment), and were part of the decision-making process in their organizations (high opportunity) were more likely to experience higher levels of job satisfaction compared to others who did not. Then, in conjunction with self-efficacy (*β* = 0.20, *p* < 0.05) and amotivation (*β* = 0.25, *p* < 0.05), job satisfaction had a significant effect on personal wellbeing (*β* = 0.33, *p* < 0.001). Remote workers with higher levels of self-efficacy, lower amotivation, and higher job satisfaction were likely to have greater personal wellbeing compared to others. 

While the relationships outlined in the SEM are also apparent in the two estimated OLS models, the SEM provides a graphical representation of the covariance between the explanatory constructs and outcomes of focus. All covariances in the SEM (amongst self-efficacy, intrinsic motivation, affective commitment, opportunity, and amotivation) were statistically significant at the *p* < 0.001 level. The model shows a positive covariance between each construct, except for the negative covariance introduced by amotivation. Therefore, remote employees with higher self-efficacy are also likely to have higher intrinsic motivation, affective commitment, and opportunity, and lowered amotivation; this pattern holds across each exploratory construct. While the direct effects suggest some constructs were more important than others in relation to job satisfaction and wellbeing, the significant covariance between constructs implies all explanatory variables in the SEM worked closely together in their effect on job satisfaction and then personal wellbeing. This demonstrates the importance of each factor *equally,* since one affects the other to produce desired outcomes. 

### 4.4. Objective (d): The Role of HR Bundles and Organizational Culture

While a clear pattern of interdependence emerged from the SEM, HR bundles and organizational culture were not identified as significant contributors to job satisfaction. Yet, the literature provides ample evidence of the importance of HR bundles, especially within the AMO context. Table 4 shows the bivariate correlations between HR bundles, culture factors, and significant explanatory variables as established in the OLS and SEM. Results in Table 4 show all HR bundles were statistically and significantly correlated with the five explanatory constructs examined in the SEM. Table 4 highlights the two strongest Pearson’s *r* coefficients in the matrix. 

At least one organizational culture construct (LMX, work culture, and teamwork) had the strongest correlation with self-efficacy, intrinsic motivation, affective commitment, opportunity, and amotivation. This finding suggests organizational culture plays an indirect but important role in job satisfaction. HR bundles were strongly correlated with the direct factors of job satisfaction. When remote workers benefitted from ability-enhancing practices (e.g., training programs), motivation-enhancing practices (e.g., bonuses), effective staffing policies (e.g., best selection in hiring), internal mobility opportunities (e.g., a path to promotion), job security, a clear job description, and regular performance appraisals, there was a decrease in their amotivation and an increase in their self-efficacy, intrinsic motivation, affective commitment, and opportunity to participate. These findings further demonstrate the interconnectivity of organizational HR practices, AMO factors, job satisfaction, and personal wellbeing. While HR bundles and organizational culture indirectly affected job satisfaction, they had a direct effect on the most important *predictors* of job satisfaction and personal wellbeing. 

**Figure 2:**
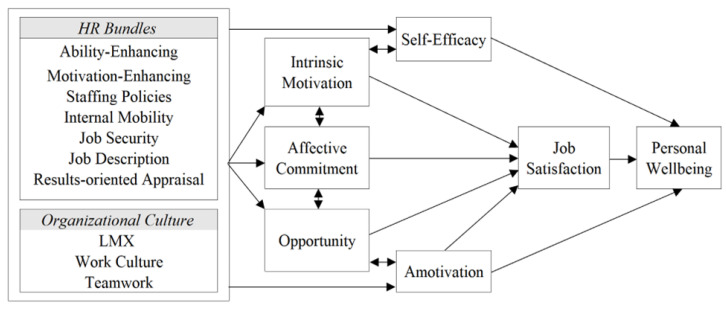
Summary of Findings.

Figure 2 summarizes the results to show the indirect and direct relationships of the constructs examined in this study. While HR bundles and organizational culture did not directly impact job satisfaction and personal wellbeing, they were correlated with the direct predictors. HR bundles and culture are influential to employees’ self-efficacy, intrinsic motivation, affective commitment, opportunity, and amotivation. Therefore, HR bundles and organizational culture should be given consideration in creating a work environment that facilitates job satisfaction and personal wellbeing. In Figure 2, intrinsic motivation, affective commitment, opportunity, and amotivation directly affect job satisfaction. While self-efficacy did not impact job satisfaction, it directly affected personal wellbeing in conjunction with amotivation and job satisfaction. 

## 5. Discussion

The purpose of this study was to examine the factors affecting employee job satisfaction and wellbeing among remote workers in Utah through the AMO conceptual lens. As employees are at the forefront of organizational success, it is important to understand key factors affecting their job satisfaction and personal wellbeing. Results relating to job satisfaction were supported by the literature [25,26,31]. A strong correlation between internal motivation and employee satisfaction, indicating those who enjoyed their work tasks experienced higher levels of satisfaction [31]. This study showed that remote workers who enjoyed their work tasks (intrinsically motivated), had opportunities to participate in decision-making processes, and felt a personal connection to their organization’s goals (affective commitment) had higher levels of job satisfaction compared to those who did not. As such, they were found to stay longer in their jobs because they wanted to, experiencing a greater sense of perceived satisfaction. 

While finding pleasure in one’s work tasks is important, another critical factor was the opportunity to participate in the organizational decision-making process. Ensuring employees are given the right environment to voice opinions and ideas was found to influence their job satisfaction. As previously highlighted, decision-making opportunities were a common characteristic of a redesigned job, specifically related to workflow decisions [31]. When employees are provided with an environment that facilitates a certain level of autonomy over their workday and workflow decisions, their sense of satisfaction increases. Furthermore, this study’s finding on the opportunity to participate was supported by the literature, which indicated that meaningful and enriching work tasks were likely to improve job satisfaction, motivation, and performance [32]. When remote employees work in a supportive and open environment where they are heard, their sense of satisfaction increases. 

A major factor found to reduce job satisfaction was amotivation. Remote workers who felt helpless or did not see the purpose of their work had lower levels of job satisfaction. This is turn can negatively affect productivity and retention rates, as employees seek better job opportunities. Redesigning work tasks can help reduce levels of amotivation among remote employees, as they have some control over the structure of their tasks. For example, a remote worker with the flexibility to restructure tasks based on their immediate environment can potentially reduce the number of nonwork intrusions in their workday. When remote workers can enrich their work tasks, their perceived sense of connection and satisfaction likely increases. Creating meaningful work tasks and roles (as perceived by an employee) can improve their overall levels of intrinsic motivation and affective commitment to the organization. Additionally, allowing remote workers the opportunity to participate in broader decision-making processes (e.g., leading projects, asking for their feedback) can help lower levels of amotivation and increase levels of satisfaction. To encourage employee retention, HRM policies should focus on activities that enhance job satisfaction attributes and lower amotivation. 

These results also appear to align well with the focus of Commitment HR systems, which center on developing positive attitudes and behaviors by making emotional connections between organizational and employee goals [21]. Moreover, Commitment HR systems are characterized by self-directed employees, organizational trust and transparency, and high employee involvement [22], all of which correspond with the findings that intrinsic motivation, affective commitment, and opportunity positively affect job satisfaction among remote workers in Utah. Commitment HR systems appear to be well-suited for organizations with remote work environments. As explained, employees must be self-directed, able to function autonomously, and operate in a collaborative work setting [31]. Coupled with activities that facilitate antecedents of job satisfaction and reduce feelings of amotivation, organizations practicing remote work using a Commitment HR system may expect lower turnover rates. If a Commitment HR system is already in place, but HRM has not adopted a remote work environment, managers should explore their organization’s compatibility with the practice [14,15,16,17]. With a supportive environment from HRM, remote employees can experience higher levels of commitment and trust in their organization’s goals.

The results also aligned with other studies’ findings, which showed that remote workers whose personal values align with those of their organization (high affective commitment) were found to stay longer and experience a greater sense of job satisfaction [68]. As such, remote workers should consider employment with organizations that share their personal values and foster positive employee–employer relationships. Additionally, HRM should recognize that while salary, benefits, bonuses, awards, and other acknowledgments are important, they do not entirely account for employee job satisfaction. Well-paid and highly praised remote workers can still experience low job satisfaction without a sense of purpose in their work tasks. As this sense of purpose stems from the alignment of personal values and proficiency in their roles, HRM should invest in professional development and culture training for remote workers, which will help them feel a greater sense of purpose and job satisfaction. These activities also complement redesigning work tasks. 

This study also found self-efficacy and job satisfaction had positive effects on personal wellbeing, while amotivation had negative effects. Remote workers who experienced higher levels of personal wellbeing were more confident in their ability to perform their work tasks. HRM seeking to positively influence the personal wellbeing of remote workers could invest in professional development opportunities that target competencies related to their job functions. This type of activity is also consistent with Commitment HR systems [22]. When remote workers experience high levels of self-esteem and are proficient in their work tasks, they are more likely to experience higher levels of personal wellbeing. Conversely, when remote workers experienced a lower sense of purpose in their role, their personal wellbeing was negatively affected. As previously defined, personal wellbeing in this study is related to an individual’s level of psychological distress [78]. When remote workers do not see the value of their work tasks, they are likely to experience higher levels of stress. Results also found a direct relationship between job satisfaction and personal wellbeing, consistent with other findings [11]. As remote employees experienced greater job satisfaction, they were more likely to have higher levels of personal wellbeing compared to others with lower job satisfaction. This finding suggests that the direct factors of job satisfaction can indirectly affect personal wellbeing. 

As remote workers are intrinsically motivated, have opportunities to participate in decision-making processes, and feel a sense of emotional and personal commitment to their organization, their job satisfaction increases, which in turn increases their level of personal wellbeing. However, amotivation or the inability to determine the purpose of work tasks negatively affected both job satisfaction and personal wellbeing. As such, redesigning work tasks and allowing remote employees certain levels of autonomy and flexibility can help increase employee enthusiasm [12,31]. This, coupled with skills training and enhancing job satisfaction attributes, can provide a supportive and creative remote work environment that fosters employee retention. As HRM assesses their organizations’ current investment in professional development opportunities for remote workers, they can also examine whether increasing this investment would be appropriate. Providing incentives that encourage positive perceptions toward competency training programs is important, as it could help motivate employee participation. Additionally, remote workers should appraise their own level of participation in professional development opportunities offered by their employers as well as other personal learning opportunities that would improve their abilities and confidence to perform their job functions.

Findings from this study also provided strong evidence to suggest that the role of HRM had no direct effect on the personal wellbeing of remote workers. However, the results raise awareness as to how HRM can indirectly influence personal wellbeing through factors that affect job satisfaction, self-efficacy, and amotivation. Fundamentally, there is an unknown number of external factors (e.g., family circumstances, relationships, health) that affect personal wellbeing beyond the scope of remote workers’ formal employment. The literature also identified many unique challenges employees face in a remote work environment, such as work–life balance, isolation, loneliness, overworking, work–family conflict, and asynchronous communication [11,12,38,79,80]. The findings presented in this study can help HRM better understand the indirect effects of HR policies and how they might influence the factors of job satisfaction and personal wellbeing. Furthermore, HRM should not expect the mere adoption of remote work or other flexible workplace policies to improve employees’ personal wellbeing. For example, remote work policies, codes of conduct, equal opportunity, privacy, paid time off, sick leave, maternity/paternity leave, and sexual harassment may also affect the job satisfaction and personal wellbeing of remote workers. 

This study’s findings identified the significant roles of intrinsic motivation, amotivation, opportunity, and affective commitment on job satisfaction while demonstrating the direct effects of self-efficacy, amotivation, and job satisfaction on personal wellbeing. Aside from the negative effects of remote work discussed in one study [33], the results showed remote workers are most likely to experience higher levels of job satisfaction when they (a) found purpose or value in their work tasks (low amotivation), (b) experienced pleasure or excitement in their roles (high intrinsic motivation), (c) felt a personal connection to their organization (high affective commitment), and (d) were included in their organization’s decision-making process (high opportunity). These results support the AMO framework that guided this study, which explained the relationship between employee performance and HRM [40,41,42]. 

The results indicated the extent to which HRM can expect high performance from remote workers by focusing on improving job satisfaction and addressing the factors directly influencing it (intrinsic motivation, opportunity, amotivation, and affective commitment). Policies focused on aligning the roles and tasks of remote workers with their skills, interests, and values could help increase remote workers’ level of job satisfaction. In addition, providing remote workers with greater autonomy to make decisions and solve problems gives them more control and ownership over their work. Opportunities to learn new skills, take on new challenges, collaborate cross-functionally, and provide feedback to management are other meaningful ways to help remote workers feel more involved in their organization. Further, amotivation can be reduced by providing remote workers with clear goals and expectations, so they know what is required of them. Providing timely, constructive feedback can also help them understand how they are performing and what they need to do to improve their performance. However, the value of recognition and rewards should not be overestimated. HRM should acknowledge the significant contributions of top-performing employees, provide fair compensation, and engage in policies and activities that support work–life balance.

Evidence from this study confirmed findings on the positive effects of employees’ participation in the workplace (opportunity) [44]. Given the positive, direct effect of job satisfaction on personal wellbeing, remote workers are more likely to experience higher levels of personal wellbeing when they feel confident in their ability to perform their job functions (high self-efficacy) and find meaning in carrying out their job tasks (low amotivation). Remote employees who experienced higher personal wellbeing were also likely to have higher levels of intrinsic motivation, affective commitment, and opportunity, and lowered amotivation. This finding supports a connection between remote work and personal wellbeing and is consistent with other studies [11,12,13]. 

In this study, the role of HR bundles and organizational culture were examined in relation to job satisfaction and personal wellbeing. Findings showed that both HR bundles and organizational culture are indirectly related to job satisfaction. However, there was a strong correlation between HR bundles and the direct predictors of job satisfaction and personal wellbeing (self-efficacy, intrinsic motivation, affective commitment, opportunity, and amotivation). When remote workers benefitted from ability-enhancing practices (e.g., training programs), motivation-enhancing practices (e.g., bonuses), effective staffing policies (e.g., best selection in hiring), internal mobility opportunities (e.g., a path to promotion), job security, a clear job description, and regular performance appraisals, there was a decrease in their amotivation and an increase in their self-efficacy, intrinsic motivation, affective commitment, and opportunity to participate. As HRM seeks to develop a successful work environment fostering employee retention, they can consider policies focused on supporting these practices. Overall, these findings support the literature describing the importance of HR bundles within the AMO framework [47,48] and show the extent to which organizational HR practices, AMO factors, job satisfaction, and personal wellbeing are interrelated. 

A construct that was strongly correlated with the predictors of job satisfaction and personal wellbeing was LMX, work culture, and teamwork. As such, practices that foster leader–member exchange, work cultures conducive to productivity, employee motivation and participation, and teamwork are all consistent with Commitment HR systems. When management can provide positive work cultures, support employee involvement, and encourage team building, intrinsic motivation, self-efficacy, affective commitment, opportunity, and amotivation improve. This study also provides evidence highlighting the importance of designing hiring policies that identify candidates who are intrinsically motivated, a good fit for Commitment HR systems, and whose personal values and goals align with the organization. The overall results of this study add to the literature exploring job satisfaction and personal wellbeing because they establish the importance of all factors somewhat equally, as each one affects another’s capacity to yield desired outcomes. Essentially, all factors play a role in remote employees’ satisfaction and personal wellbeing. There is no lone function HRM can implement to positively influence the job satisfaction or personal wellbeing of remote workers. As HRM seeks to develop successful remote work environments, it is important to consider all factors that positively influence employees’ job satisfaction and wellbeing and the bundle of HR activities that can support these outcomes.

## 6. Conclusions

The AMO framework considered three variables—ability, motivation, and opportunity—and their role in employee performance. This study highlighted factors affecting remote worker job satisfaction and personal wellbeing, the relationships among significant predictors of job satisfaction and personal wellbeing, and the role of HR policies and organizational culture in remote work environments. Overall, remote employees who saw purpose or value in their work tasks (low amotivation), experienced pleasure or excitement in their roles (high intrinsic motivation), felt like they had an emotional attachment and personal connection to the organization (high affective commitment), and were part of the decision-making process in their organizations (high opportunity) were more likely to experience higher levels of job satisfaction. Additionally, remote employees who were confident in their abilities to perform job tasks (high self-esteem), felt a sense of purpose in their work (low amotivation), and experienced high levels of job satisfaction tended to experience higher levels of personal wellbeing.

When considering the relationships among predictors of job satisfaction and personal wellbeing, all predictors affected job satisfaction first, then personal wellbeing. This implies that remote employees may experience high levels of job satisfaction first, then personal wellbeing. The results of this research also underline the importance of HR practices and organizational culture in employee job satisfaction and wellbeing within remote work. Organizational culture played an indirect, but important role in job satisfaction. HR bundles were strongly correlated with the direct predictors of job satisfaction, and in turn, job satisfaction was found to directly influence personal wellbeing. The study highlights HR practices that could support worker satisfaction and wellbeing and reduce feelings of amotivation. Overall, when remote workers benefitted from ability-enhancing practices (e.g., training programs), motivation-enhancing practices (e.g., bonuses), effective staffing policies (e.g., best selection in hiring), internal mobility opportunities (e.g., a path to promotion), job security, a clear job description, and regular performance appraisals, there was a decrease in amotivation levels and an increase in self-efficacy, intrinsic motivation, affective commitment, and opportunity to participate.

Also explained by the AMO framework were high-performance work systems (HPWS) and the responsibility of HRM to create such systems. It is important to note that while HR bundles and organizational culture indirectly affected job satisfaction, they had a direct effect on the most important predictors of job satisfaction and personal wellbeing. An effective HPWS supports employees’ skills, incentives to perform, and opportunities to contribute discretionary effort. Practices that enhanced these dimensions were referred to as high-performance work practices (HPWPs). Based on the findings of this study, HRM should consider HPWPs that support the factors affecting job satisfaction and personal wellbeing, which in turn will foster an effective HPWS. Practices include skill-building training programs, clear job expectations and descriptions, opportunities for advancement and participation, clear paths to promotion, best-fit recruitment and selection processes, regular performance appraisals, job security, and bonuses. Furthermore, Commitment HR systems and work redesign should also be important considerations for HRM, as they have implications for positively influencing affective commitment, HPWPs, and ultimately, HPWS. Within such a system, employee turnover is expected to decrease over time. 

## 7. Recommendations and Implications

Based on the results of this study, the authors provide several recommendations for HR professionals and remote workers. These practices can be considered as HPWPs that will support a HPWS. Our first recommendation is to recognize the value in having remote workers feel a sense of purpose in their work and advocate for the alignment of personal values with organizational values. In symmetry with this recommendation, hiring policies should clearly define and describe organizational values. This includes prioritizing hiring remote workers who are intrinsically motivated and whose personal values and goals align with those of the organization. This can be done by asking specific motivation questions during the interview process or designing a specialized assessment tool. Once hired, such employees will be more likely to stay with the organization if it adopts a Commitment HR system. Commitment HR systems focus on developing positive attitudes and behaviors through emotional connections between organizational and employee goals. 

Another recommendation is to encourage remote workers to define their own personal values and understand the values of their current and future potential employers. Remote workers should familiarize themselves with the characteristics of Control and Commitment HR systems as they navigate their careers and consider their personal preferences and compatibility with each system type. Remote workers should seek employment with organizations that share their personal values to increase their likelihood of job satisfaction. While compensation and recognition are important, they cannot (by themselves) guarantee job satisfaction. 

Third, we recommend that *HR* invests in professional development and culture training for remote workers. These types of investments help employees feel a greater sense of purpose in their work, thereby increasing the probability of job satisfaction. The results of this study suggest a direct relationship between self-efficacy and personal wellbeing among remote workers. Remote workers who are confident in their abilities to perform their work well also experience higher levels of personal wellbeing. HR professionals should invest in professional development opportunities that target skills related to remote workers’ job functions to positively influence their personal wellbeing. 

Remote workers should appraise their own level of participation in professional development and training opportunities offered by their employers, as well as other personal learning opportunities that would improve their abilities and confidence in performing their job functions. This also means that *HR* professionals should assess their organization’s current investment in professional development opportunities for remote workers and consider whether increasing such investments might be appropriate. It is also important for HR professionals to recognize that while HR bundles and organizational culture can indirectly influence job satisfaction, they may not have a direct impact on personal wellbeing. It is important to consider external factors that may affect remote workers’ wellbeing and provide appropriate support and resources, when possible.

Next, organizations should provide regular opportunities for remote workers to participate and be included in organizational decision-making processes. HR professionals can provide remote workers with greater autonomy by offering opportunities to learn new skills, take on new challenges, collaborate cross-functionally, and provide feedback to management. This study reinforces earlier research that suggests that these elements help to improve job satisfaction. Offering bonuses and other incentives to recognize the contributions of top-performing remote workers should be considered. HR managers can also improve job satisfaction and personal wellbeing by seeking feedback and input from remote workers and being intentional about including them in the organization’s decision-making processes.

Finally, there is a need to educate HR professionals about the unique challenges that remote workers face, such as isolation, loneliness, overwork, and work–family conflicts. A solid understanding of these challenges, as well as what work–life balance means to remote workers, can inform the creation of HR bundles that help workers cope with ongoing challenges. This may require redesigning work tasks and adjusting levels of autonomy to better accommodate the unique challenges of working remotely. This study demonstrates that HR policies and organizational culture have an indirect effect on the personal wellbeing of remote workers through its impact on job satisfaction. Our study provides insights that can assist organizations and remote employees in navigating the decisions and complexities of sustaining an effective and efficient remote work environment. This research, along with other relevant literature, can guide HRM’s efforts to improve remote workers’ satisfaction and personal wellbeing.

## Figures and Tables

**Table 1:** Descriptive Summary and Reliability of Constructs after CFA.

Domain	Construct	Min.	Max.	Mean	*SD* ^1^	A ^2^	S ^3^	K ^4^
Ability (A)	Self-Efficacy	1	100	82.64	15.77	0.92	1.16	1.14
Motivation (M)	Intrinsic Motivation	1	5	3.68	0.99	0.89	0.64	0.04
	External Regulation	1	5	3.76	0.94	0.85	0.79	0.32
	Identified Regulation	1	5	3.95	0.87	0.82	1.08	1.42
	Amotivation	1	5	2.07	1.02	0.90	0.58	0.84
Opportunity (O)	Opportunity	1	5	3.56	0.83	0.86	0.42	0.20
HR-Bundles (Policies)	Ability-Enhancing	1	5	3.43	1.01	0.89	0.35	0.71
	Motivation-Enhancing	1	5	3.16	0.95	0.85	0.30	0.82
	Staffing Policies	1	5	3.75	0.84	0.89	0.52	0.08
	Internal Mobility	1	5	3.57	0.88	0.74	0.18	0.52
	Job Security	1	5	3.93	0.85	0.77	0.59	0.24
	Job Description	1	5	3.83	0.86	0.89	0.47	0.58
	Results-Oriented Appraisal	1	5	3.63	0.79	0.80	0.21	0.28
Organizational Culture	LMX: Supervisor Relationship	1	7	5.98	1.01	0.92	1.14	0.78
	Work Culture	1	7	5.84	1.09	0.94	1.16	0.85
	Teamwork	1	5	4.15	0.75	0.90	1.20	2.00
Organizational Commitment	Affective Commitment	1	7	4.79	1.63	0.91	0.44	0.77
	Continuance Commitment	1	7	5.52	1.08	0.81	1.05	1.21
	Normative Commitment	1	7	4.07	1.44	0.89	0.02	0.69
Outcome 1	Job Satisfaction	1	7	4.76	1.44	0.90	0.55	0.28
Outcome 2	Personal Wellbeing: GHQ-12	1	5	3.57	0.72	0.90	0.27	0.20

Note: ^1^ Standard Deviation; ^2^ Cronbach’s alpha; ^3^ Absolute Skewness; ^4^ Absolute Excess Kurtosis.

**Table 2:** Bootstrapped Regression of Selected Factors on Job Satisfaction.

Factors	β	Bias	SE	*p*	95% Confidence Interval [BC]	
					Lower	Upper
Income ^1^	−0.03	0.00	0.07	0.60	−0.18	0.10
Self-Efficacy	0.00	0.00	0.01	0.52	−0.01	0.02
Intrinsic Motivation	0.47	0.00	0.14	0.00 ***	0.21	0.74
External Regulation	−0.09	0.00	0.08	0.23	−0.24	0.05
Identified Regulation	−0.12	0.02	0.15	0.43	−0.44	0.24
Amotivation	−0.39	0.00	0.12	0.00 ***	−0.63	−0.15
Opportunity	0.39	−0.01	0.15	0.01 *	0.10	0.67
Ability-Enhancing	0.06	0.01	0.09	0.54	−0.15	0.26
Motivation-Enhancing	0.13	−0.01	0.11	0.22	−0.07	0.33
Staffing Policies	−0.19	0.00	0.12	0.11	−0.43	0.04
Internal Mobility	0.00	0.00	0.11	1.00	−0.24	0.23
Job Security	−0.09	0.00	0.10	0.37	−0.28	0.10
Job Description	−0.09	0.00	0.13	0.50	−0.35	0.16
Results-Oriented Appraisal	−0.01	0.00	0.15	0.97	−0.29	0.28
LMX (Supervisor Relationship)	0.24	0.00	0.14	0.10	0.00	0.52
Work Culture	−0.20	0.00	0.12	0.11	−0.47	0.03
Teamwork	0.19	0.00	0.17	0.27	−0.13	0.53
Affective Commitment	0.22	−0.01	0.08	0.00 ***	0.07	0.35
Continuance Commitment	−0.10	0.00	0.08	0.22	−0.27	0.08
Normative Commitment	0.05	0.00	0.06	0.41	−0.06	0.15

Note: * *p* < 0.05, ** *p* < 0.01, *** *p* < 0.001. ^1^ Income included in the model for control only.

**Table 3:** Bootstrapped Regression of Selected Factors on Personal Wellbeing.

Factors	β	Bias	SE	*p*	95% Confidence Interval [BC]	
					Lower	Upper
Income ^1^	0.05	0.00	0.06	0.38	−0.05	0.17
Self-Efficacy	0.01	0.00	0.01	0.00 ***	0.00	0.02
Intrinsic Motivation	−0.02	−0.02	0.12	0.85	−0.27	0.16
External Regulation	0.08	0.00	0.06	0.15	−0.03	0.18
Identified Regulation	−0.12	0.01	0.10	0.24	−0.33	0.10
Amotivation	−0.15	0.00	0.07	0.04 *	−0.30	0.00
Opportunity	−0.11	0.01	0.10	0.29	−0.31	0.14
Ability-Enhancing	−0.01	0.01	0.07	0.91	−0.16	0.15
Motivation-Enhancing	−0.06	0.01	0.07	0.36	−0.20	0.09
Staffing Policies	0.12	−0.01	0.09	0.16	−0.04	0.28
Internal Mobility	0.07	0.00	0.09	0.42	−0.12	0.25
Job Security	−0.03	−0.01	0.08	0.69	−0.18	0.09
Job Description	−0.04	0.01	0.09	0.67	−0.22	0.18
Results-Oriented Appraisal	0.02	−0.01	0.11	0.86	−0.17	0.21
LMX (Supervisor Relationship)	0.03	0.01	0.09	0.72	−0.14	0.24
Work Culture	0.03	0.00	0.08	0.67	−0.12	0.17
Teamwork	0.05	−0.01	0.11	0.64	−0.15	0.24
Affective Commitment	−0.11	0.01	0.06	0.06	−0.23	0.02
Continuance Commitment	−0.05	0.00	0.05	0.29	−0.13	0.03
Normative Commitment	0.03	−0.01	0.05	0.55	−0.06	0.10
Job Satisfaction	0.26	0.00	0.08	0.00 ***	0.10	0.41

Note: * *p* < 0.05, ** *p* < 0.01, *** *p* < 0.001. ^1^ Income included in the model for control only.

**Table 4:** Zero-Order (Pearson’s) Correlations between HR Factors and SEM Predictors.

	Self-Efficacy	Intrinsic Motivation	Affective Commitment	Opportunity	Amotivation
Ability-Enhancing	0.3 5***	0.36 ***	0.46 ***	0.44 ***	−0.32 ***
Motivation-Enhancing	0.18 *	0.18 *	0.31 ***	0.46 ***	−0.25 **
Staffing Policies	0.32 ***	0.40 ***	0.55 ***	0.65 ***	−0.40 ***
Internal Mobility	0.30 ***	0.38 ***	0.54 ***	0.52 ***	−0.48 ***
Job Security	0.26 **	0.24 **	0.42 ***	0.50 ***	−0.35 ***
Job Description	0.42 ***	0.30 ***	0.39 ***	0.50 ***	−0.36 ***
Results-Oriented Appraisal	0.41 ***	0.29 ***	0.48 ***	0.55 ***	−0.31 ***
LMX: Supervisor Rel.	0.50 ***	0.42 ***	0.47 ***	0.46 ***	−0.46 ***
Work Culture	0.47 ***	0.45 ***	0.54 ***	0.62 ***	−0.45 ***
Teamwork	0.43 ***	0.45 ***	0.58 ***	0.61 ***	−0.51 ***

Note: * *p* < 0.05, ** *p* < 0.01, *** *p* < 0.001.

## Data Availability

The data presented in this study are available on request from the corresponding author. The data are not publicly available due to ethical protocols outlined by the Institutional Review Board.

## Appendix A

**Table A1:** Description of Constructs.

Domain	Construct (No. of Items)	Interpretation	Original Scale
Ability (A)	Self-Efficacy (8)	One’s self-assessed capability to execute a behavior to produce certain outcomes.	[81]
Motivation (M)	Intrinsic Motivation (4)	Performing an activity for pleasure and satisfaction inherent in the activity itself.	[82]
	External Regulation (4)	Performing an activity for external rewards or to avoid negative consequences.	
	Identified Regulation (4)	Performing an activity because it is perceived as valued/important and self-chosen.	
	Amotivation (4)	Performing an activity even though there is no sense of perceived purpose or expectation of its value.	
Opportunity (O)	Opportunity (4)	One’s level of participation in the decision-making process of the organization, measured as ‘Employee Involvement’.	[48,83]
HR Bundles (Policies)	Ability-Enhancing (5)	HR policies to improve employees’ ability to perform in their roles (e.g., training opportunities).	[83]
	Motivation-Enhancing (6)	HR policies to reward employees based on performance (e.g., internal rewards, bonuses).	
	Staffing Policies (4)	HR policies to screen and select the ‘right’ candidate for the role (e.g., structured interviews).	[84]
	Internal Mobility (3)	HR policies that facilitate an internal promotion process for the upward mobility of employees (e.g., career ladder).	
	Job Security (2)	HR policies that provide employees with a sense of longevity and security in the role (e.g., long-term contracts).	
	Job Description (3)	HR policies that provide employees with a clear and up-to-date description of their responsibilities.	
	Results-Oriented Appraisal (3)	HR policies that enable a fair and objective performance review system (e.g., annual reviews).	
Organizational Culture	LMX: Supervisor Relationship (8)	Perceived quality of the relationship between an employee and their immediate supervisor (adaptation of the LMX-7).	[85]
	Work Culture (9)	Perceived level of cohesion, support, and openness between organizational leaders and employees (adaptation of the Corporate Culture Scale—Short Form).	[86]; Original German version—[87]
	Teamwork (6)	Perceived level of teamworking between employees to effectively attain desired performance outcomes (adaptation of the Internal Participation Scale).	[88]
Organizational Commitment	Affective Commitment (5)	One’s emotional attachment to the goals and values of the organization (i.e., ‘want to stay’).	[68]
	Continuance Commitment (7)	One’s perception towards the personal cost of leaving the organization (i.e., ‘need to stay’).	
	Normative Commitment (6)	One’s perception of their moral obligation to stay with the organization (i.e., ‘ought to stay’).	
Outcome 1	Job Satisfaction (5)	One’s general level of satisfaction with their employment (Short Index of Job Satisfaction—SIJS)	[89]
Outcome 2	Personal Wellbeing (12)	A measure of an individual’s level of psychological distress based on the GHQ-12.	[78]
